# Reassortment of bluetongue virus vaccine serotypes in cattle

**DOI:** 10.4102/jsava.v89i0.1649

**Published:** 2018-12-05

**Authors:** Carien van den Bergh, Peter Coetzee, Estelle H. Venter

**Affiliations:** 1 Department of Veterinary Tropical Diseases, University of Pretoria, South Africa; 2 Equine Research Centre, University of Pretoria, South Africa; 3 College of Public Health, James Cook University, Australia

## Abstract

Bluetongue is primarily a disease of sheep in South Africa, while cattle and goats are mostly subclinically infected. The viraemia of bluetongue virus in cattle lasts much longer than in sheep and the role of cattle in the epidemiology of bluetongue in South Africa is poorly understood. Bluetongue virus has a segmented double-stranded ribonucleic acid genome and reassortment of genomes is a common feature. The aim of the study was to investigate whether reassortment occurs between vaccine and field strains when simultaneously administered to cattle. Six cattle between the ages of 6 and 12 months were infected with five strains of modified live vaccine bluetongue virus and a virulent field isolate of bluetongue virus 4. Blood samples were subsequently collected daily from these animals from day 1 to day 39 post-inoculation. Viruses were directly isolated during viraemia from the buffy coat on Vero cells using the plaque forming unit method. Analysis of plaques indicated that no reassortants between virulent field and vaccine strains occurred and the virulent bluetongue virus 4 was identified as the predominant virus strain. However, a reassortant virus between two bluetongue virus vaccine strains was isolated from the buffy coat. Whole genome sequences from the vaccine viruses were compared to the suspected reassortant and it was found that segment 8 exchanged between the bluetongue virus 8 and bluetongue virus 9 vaccine strains. The use of the live-attenuated bluetongue virus multivalent vaccine in South Africa causes circulation of different vaccine serotypes in *Culicoides* spp. and susceptible hosts and cattle might provide the ideal host for reassortment to occur.

## Introduction

**FIGURE 1:**
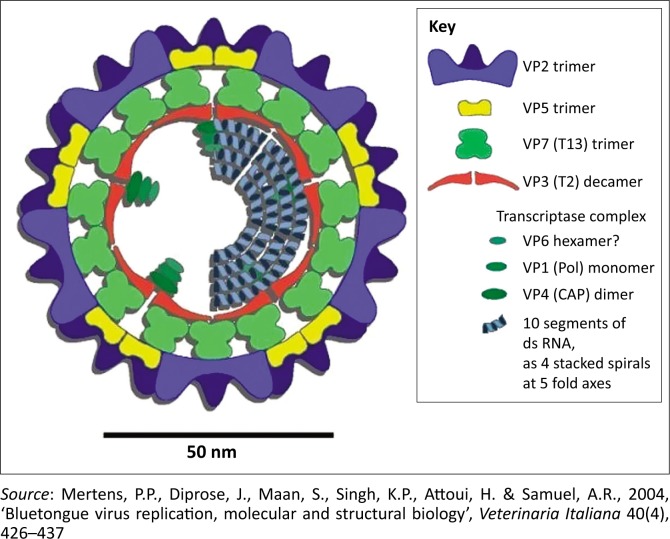
Schematic representation of bluetongue virus illustrating the interactions of the different proteins. VP, viral proteins; RNA, ribonucleic acid; CAP, capping enzyme; Pol, polymerase.

Bluetongue (BT) is a non-contagious viral disease of domestic and wild ruminants with serious socio-economic impacts. Infection with bluetongue virus (BTV), the causative agent, results in clinical disease in cattle and sheep, including reproduction losses and consequently international movement restriction of ruminants (Howell & Verwoerd 1971). Bluetongue virus is an *Orbivirus* that belongs to the family Reoviridae. The virus genome consists of 10 segments of double-stranded ribonucleic acid (dsRNA) that codes for seven structural viral proteins (VPs: VP 1 – VP 7) and five non-structural proteins (NS) as illustrated in Figure 1 (NS 1, NS 2, NS 3/3a and NS 4) (Mertens et al. 2004). Structural VP 3 encoded by segment 3 forms the shell of the sub-core of the virus. The outer layer of the virion consists of VP 2 encoded by segment 2. The function of VP 1, the RNA-dependent RNA polymerase encoded by segment 1, is transcription and replication (Boyce et al. 2004). Structural VP 4 caps the newly synthesised messenger RNA (capping and trans-methylase enzyme, encoded by segment 4) (Ramadevi et al. 1998) and VP 6 (encoded by segment 9) unwinds and re-anneals dsRNA during transcription and replication (RNA-dependent ATPase and helicase, encoded by segment 9) (Stauber et al. 1997). Structural VP 7 is a ligand for the insect cell receptor and appears to be able to mediate the attachment between BTV and the membrane proteins of the cells (Basak et al. 1997). The outer capsid proteins are involved in attachment (Hassan & Roy 1999) and release (VP 5) of the virus into the cytoplasm of mammalian cells (Hassan et al. 2001).

The non-structural protein NS 1 (encoded by segment 5) is the most abundant in the infected cells and forms tubular structures in the cell cytoplasm; the NS 1 is also a positive regulator of the virus’s expression that increases protein synthesis (Boyce, Celma & Roy 2012). Non-structural protein 2 (encoded by segment 8) assembles the proteins and nucleic acids into the mature virion (Kar, Bhattacharya & Roy 2007). Non-structural protein 3 and the alternative non-structural protein NS3a (encoded by segment 10) facilitate the release of the virus from the infected cell. The proteins also function as a bridging mechanism of VP 2 and the components of the cytoskeleton. The viral particles are released by means of either lysis of infected cells or by budding from the cell membrane (Hyatt, Zhao & Roy 1993). Non-structural protein 3 acts as an antagonist for type I interferon, an antiviral cellular response that is produced *in vitro* and *in vivo* when the host is infected with BTV (Chauveau et al. 2013). Non-structural protein 4 (encoded by segment 9) modulates the hosts’ immunity by inhibiting the transcription of interferon genes. This protects the virus and gives it enough time to replicate and spread throughout the host (Ratinier et al. 2011).

There are currently 27 BTV serotypes described worldwide, with 21 serotypes endemic to South Africa (SA) (Gerdes 2004). Bluetongue virus is transmitted between vertebrate hosts by the bite of *Culicoides* midges (Diptera: Ceratopogonidae) and the occurrence of the disease depends on the presence and the abundance of competent vectors (Mellor, Boorman & Baylis 2000).

In SA, BT is primarily a disease of sheep and controlled by annual vaccination using a freeze-dried polyvalent modified live vaccine (MLV) (Reg. No. G 358 Act No 36/1947) produced by Onderstepoort Biological Products (Pty) Ltd. The vaccine consists of three bottles (A, B, C) containing five BTV strains each: Bottle A includes BTV serotypes 1, -4, -6, -12 and -14; Bottle B includes serotypes 3, -8, -9, -10 and -11; Bottle C includes serotypes 2, -5, -7, -13 and -19 (Dungu, Gerdes & Smit 2004).

Cattle and goats are mainly subclinically infected and are not vaccinated. Cattle have a prolonged viraemia when compared to any other domestic ruminant and the virus has been isolated for up to 63 days post-infection (MacLachlan et al. 1994). Viral RNA could be detected for up to 180 days using polymerase chain reaction (PCR) (MacLachlan et al. 1994). The prolonged viraemia lends itself to extended virus replication resulting in maintaining the viral–vector cycle and viral reassortment. Reassortment is complicated further when an animal is infected with more than one strain at the same time. During reassortment, no bias towards specific segments is observed, but bias can be observed in the frequency of reassortment of specific segments when compared to other segments (Ramig et al. 1989; Samal et al. 1987a, 1987b). Vaccine virus strains can be detected in vaccinated animals with titre levels sufficient to be transferred to unvaccinated animals via *Culicoides* (Elia et al. 2008). The vaccine and wild-type strains can also exchange genome segments when simultaneous infection of an animal or *Culicoides* midge occurs, resulting in the possible emergence of viruses, for example, with altered capability to be spread by the vector in the field (Savini et al. 2008). When live-attenuated vaccine strains circulate in the field for prolonged periods, reversion to virulence is also evident by genetic drift (Veronesi, Hamblin & Mellor 2005).

Reassortment of vaccine strains was also demonstrated during BT outbreaks in 2008 in Europe. The circulation of a BTV 6 MLV strain was detected for the first time in north-western Europe in the eastern Netherlands and later in adjacent parts of Germany in cattle that displayed mild non-specific clinical signs of BT. Whole genome sequencing confirmed that the majority of genome segments of this strain were closely related to the SA BTV 6 MLV strain. The analysis also revealed that the virus had received its segment 10 (NS3/A) from an SA BTV 2 MLV strain (Maan et al. 2010). In Italy, a reassortant that contained a segment 2 (VP2) that was derived from an SA BTV16 MLV strain and a segment 5 (NS1) that was derived from an SA BTV 2 MLV strain was isolated from the field in 2002. This MLV strain was used in Italy since 2002 and annually in a multivalent vaccine in Israel since 1995 (Batten et al. 2008). Reversion to virulence of vaccine strains was also recently reported for African horse sickness virus (AHSV), a closely related *Orbivirus*, in SA (Weyer et al. 2017).

In this study, the role of cattle as hosts of BTV reassortment was investigated by the simultaneous infection of cattle with a virulent and vaccine strain of BTV.

## Materials and methods

Cattle without BTV antibodies were purchased and transported to the Faculty of Veterinary Science and housed in insect-free stables at the University of Pretoria’s Biological Research Centre. Animals were housed for a period of 2 weeks to adjust to the new food, climate and surroundings before the trial started. Both animal ethics and Section 20 approvals were obtained for the study.

Cattle aged between 6 and 12 months were subcutaneously infected with 5 MLV BTV serotypes (Bottle B, BT vaccine, Onderstepoort Biological Products, Onderstepoort, SA). The titres of the different virus serotypes in the vaccine are not indicated but according to Modumo and Venter (2012), the minimum titre per serotype is generally 5 × 10^4^ Plaque forming units/mL.

Three animals were simultaneously infected intravenously with a virulent BTV strain at a titre of 10^5.8^ per mL TCID_50_ (1 mL). This virus was previously isolated from ethylenediaminetetraacetic acid-containing blood (EDTA) taken from a clinically sick ewe. All viruses were characterised by whole genome sequencing before infection (Van den Bergh et al. 2016).

Heparinised blood samples and sera were collected daily from day 0 to day 39 post-infection. Serum samples collected at post-infection days 13, 15, 17, 19 and 21 were tested for the presence of BTV group-specific antibodies, using a commercially available competitive Enzyme-linked immunosorbent assay (ELISA) (Veterinary Medical & Research Development, Centurion, SA). Appetite and rectal temperatures of the animals were monitored daily.

Isolation and characterisation of plaques from the collected blood samples (buffy coat) were performed according to the methodology of Howell et al. (1960). Total RNA was extracted from cell culture–isolated viruses and controls by using the Trizol^®^ method according to the method prescribed by the manufacturer (Life Technologies, Fairlands, Johannesburg, South Africa). Single-stranded RNA was removed from the total RNA by precipitation with 2 M lithium chloride (Attoui et al. 2000). Ribonucleic acid obtained from isolated viruses, the vaccine parental strains from Bottle B and the wild BTV 4 strain used as controls, was analysed using polyacrylamide gel electrophoresis (PAGE). Electrophoresis was carried out using TGX™ 12% precast gels (Bio-Rad, Rosebank, Johannesburg, South Africa) at 120 V and a current of 30 mA. The running time was 6 hours and 15 minutes. Immediately following electrophoresis, the gels were stained with ethidium bromide (stock 10 mg/mL) (Invitrogen, Fairland, Johannesburg, South Africa) and gently shaken for 20 min with a bench waver shaker (MIDSci, St. Louis, United States). The gels were visualised with a Chemdoc XRS (Bio-Rad).

Whole genome sequencing (Illumina, Rosenpark, Cape Town, South Africa) was performed on control and 11 possible reassortant samples as identified by PAGE and using full-length amplification of complementary DNA as previously described by Potgieter et al. (2009). Sequence data were analysed using Geneious version 8.1.6 (Geneious R8). Neighbour-joining trees for each genome segment were generated to compare segments of each isolate with each other and to the vaccine strains. The phylogenetic trees were rooted using genome segments from AHSV as an outlier. Statistical support for each node of the phylogenetic tree was calculated using bootstrap analysis (1000 replicates).

### Ethical considerations

Animal ethics clearance was obtained from the Ethics Committee of the University of Pretoria for the experimental work. Section 20 approval according to the Animal Disease Act, 1984, was obtained from the Department of Agriculture, Forestry and Fisheries. Animals were only released from the insect-free stables when virus could not be isolated from the collected blood as stipulated by the Department of Agriculture, Forestry and Fisheries.

## Results

Daily monitoring revealed that two of the six cattle had slightly elevated temperatures of 39.1 °C and 39.4 °C on day 1 post-inoculation. Both the animals had no appetite on that specific day but returned to normal by day 2 post-inoculation. No further signs of distress were noted for any of the animals throughout the remainder of the trial. Blood samples were collected daily from the animals from day 1 to day 39 post-inoculation and viraemia was detected between day 2 and day 32 in four of the animals and in the two other animals viraemia could be detected until 39 days post-inoculation using virus isolation. Seroconversion was confirmed using a commercially available competitive ELISA (Veterinary Medical Research and Development, Centurion, SA); all cattle tested negative on day 0 and seropositive on days 13, 15, 17, 19 and 21.

**FIGURE 2:**
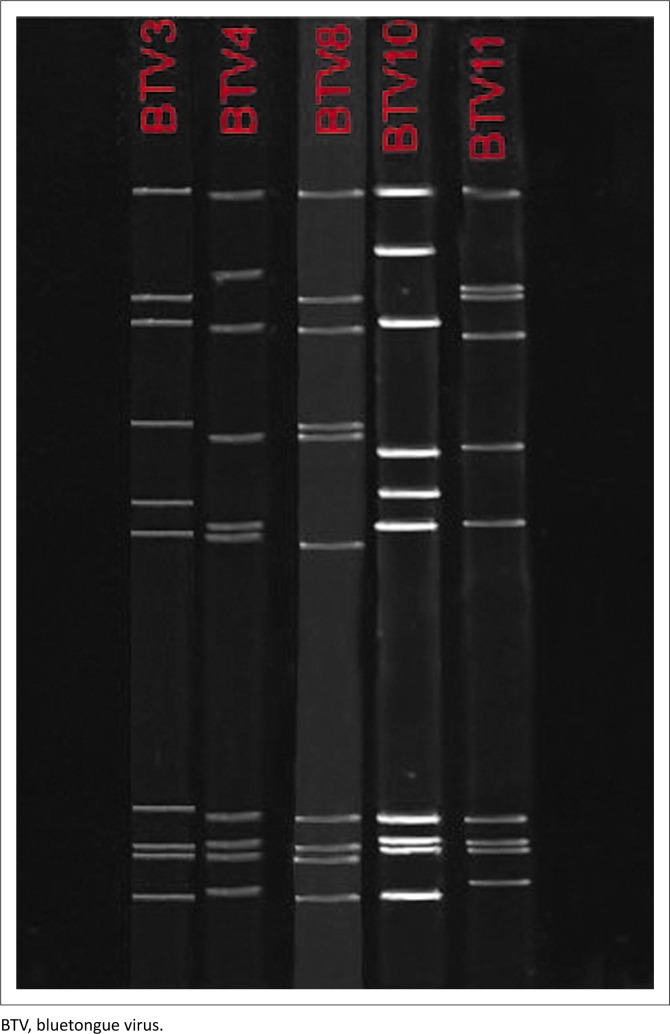
Electrophoretic profiles of the parental viruses using polyacrylamide gel electrophoresis. All 10 segments are visible and each serotype has a unique profile.

Parental strains from the vaccine bottle and the BTV 4 field strain each demonstrated unique electrophoretic profiles and it was possible to distinguish between genome segments based on their PAGE migration profiles as illustrated in Figure 2. Eleven samples were identified as possible reassortant viruses. Full-length sequences of all these samples were submitted to GenBank (Bioproject number: PRJNA287219) and sequence data (with accession numbers) were published by Van den Bergh et al. (2016).

**FIGURE 3:**
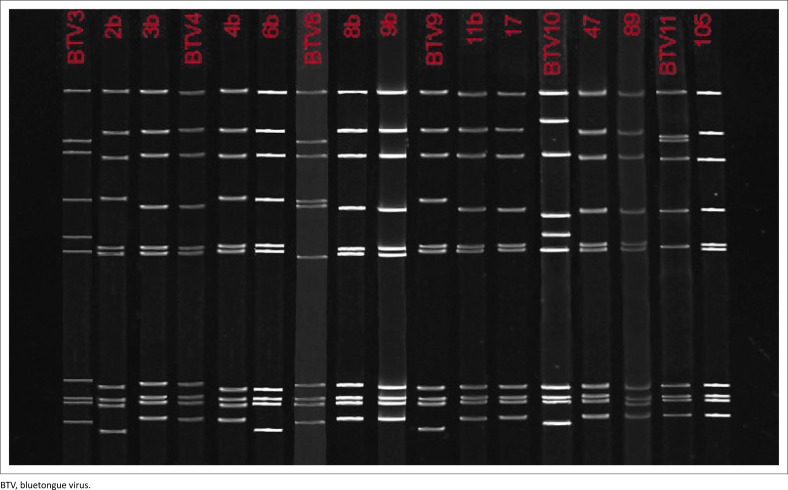
Double-stranded ribonucleic acid of plaque-purified viral samples directly from the buffy coat of animal numbers 1, 4 and 5 compared to the parental viruses. The majority of the sample resembles bluetongue virus serotype 4, while viruses 2b and 6b resemble bluetongue virus serotype 9.

**FIGURE 4:**
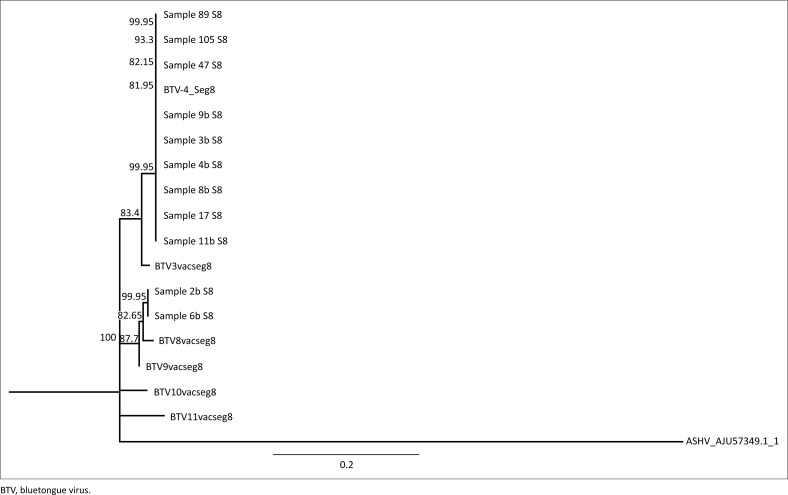
Neighbour-joining tree constructed using nucleotide sequence data of segment eight sequences of the 11 suspected reassortant isolates and the six parental viruses. Samples 2b and 6 illustrate a genome segment shift as they group with bluetongue virus vaccine strain 8.

Comparison of genomic sequences of each of the suspected reassortant isolates (*n* = 11) with the vaccine strains and the BTV 4 wild-type revealed that nine of the 11 samples analysed clustered with the wild-type BTV 4 strain in which all 10 segments were identical to the 10 segments of BTV 4. Two of the viral isolates obtained from blood samples (samples 2b and 6b, Figure 3), however, grouped with the BTV 9 vaccine strain. Nine of the segments of these isolates clustered with BTV MLV 9 with segment 8 as an exception that clustered with another vaccine strain from Bottle B, BTV 8 (Figure 4).

## Discussion

Cattle in SA are normally not affected by BTV outbreaks. Clinical signs have been reported in only two outbreaks of BT in cattle: in 1996–1997 in the Delareyville District in the North-West Province and in Donkerhoek in Bronkhorstspruit District, Gauteng Province (Barnard, Gerdes & Meiswinkel 1998). The prevalence of BTV in cattle in SA is, however, not known. To obtain BTV antibody negative cattle for this study and in a recent study by Steyn et al. (2015), 2168 cattle were bled in different geographical regions in SA (Gauteng, Mpumalanga and North-West Province) and a total of 96.5% of the cattle tested positive to BTV antibodies. It is therefore clear that although cattle have a high infection prevalence, they do not normally show any clinical signs and consequently are also not, in general, vaccinated against BTV in SA. It can therefore be concluded that cattle can be considered amplifying or maintenance hosts of the virus (MacLachlan et al. 1994). The trial was conducted during winter when midge numbers decrease significantly, therefore minimising the risk of natural infection with BTV.

During this trial, cattle showed no adverse effects of stress, with the exception of two of the cattle that were inoculated with the vaccine and the wild-type virus and developed a mild fever and loss of appetite for 1 day. Animals were viraemic from 2 to 39 days post-infection. No virus could be detected in four animals after day 33 post-infection using virus isolation, but two animals were viraemic up to 39 days post-infection. Only one of the animals with the prolonged viraemic period showed some mild signs as discussed earlier. This is in agreement with previous studies in which it has been reported that cattle can be viraemic for periods between 14 and 100 days post-infection (Sellers & Taylor 1980; Singer, MacLachlan & Carpenter 2001).

When the isolated viruses were compared to each other and to the vaccine strains using PAGE, the majority of the samples were similar to BTV 4 the wild-type strain. This might be because BTV 4 was inoculated intravenously resulting in high titres of BTV 4 in the blood compared to the vaccine serotypes that were inoculated subcutaneously according to the guidelines of the manufacturer. Another reason might be that the fastest multiplying viruses showing plaques on cell cultures were preferentially selected; thus, the virulent field strain was isolated rather than the attenuated vaccine strains (Dungu et al. 2004).

This study illustrated the generation of reassortant viruses between two vaccine strains of BTV in cattle. Segment 8 of MLV BTV 8 reassorted with MLV BTV 9. Segment 8 codes for the NS 2 which is mainly responsible for assembly of the virion (Kar et al. 2007). It can therefore be hypothesised that the reassortant virus described in this study may replicate faster to facilitate survival. Reassortment is not limited to vaccine serotypes or closely related serotypes. Maan et al. (2012) illustrated that reassortment can occur between unrelated viruses, for example, from the western and eastern lineages. Whole genome analysis of a serotype 2 isolate from India illustrated that segment 9 was unique and belonged to the eastern topotype, while segment 5 belonged to a western topotype. Another example from India was the reassortment between a field isolate of BTV 21 and 16. Whole genome sequencing confirmed segment 6 of BTV 21 clustered with BTV 16 and showed a 97.6% similarity (Shafiq et al. 2013). A study in 1987 illustrated reassortment between two field virus isolates after a Holstein Bull was inoculated with BTV 11 and 17. The isolated viruses were compared with electrophoretic profiles and 16 unique profiles were identified (Stott et al. 1987). More recent studies in Europe illustrated how BTV 16, a field isolate from Italy, and BTV 2 vaccine strain exchanged segment 5 (Batten et al. 2008) and the circulation of a BTV 6 MLV strain was detected in the eastern Netherlands and later in parts of Germany in cattle that displayed mild non-specific clinical signs of BT (Maan et al. 2010).

Whole genome sequences were performed on 116 BTV isolates from Europe, the Mediterranean region and African countries collected during 1958–2012. The isolates were compared to four MLV serotypes and compared to the 26 serotypes available on GenBank, which revealed reassortment between field isolates and vaccine strains as well as reassortment between field isolates (Nomikou et al. 2015).

The impact of BTV reassortment on the epidemiology of the disease in SA has not been studied, but reported studies indicated that it is possible that new emerging viruses can infect novel species (Purse et al. 2005). In a recent study by Weyer et al. (2017), it was confirmed that individual outbreaks of African horse sickness (caused by AHSV) in the Western Cape Province, SA, were caused by virulent revertants of the virus. Outbreaks were caused by AHSV serotype 1 live-attenuated vaccine and reassortants with genome segments derived from AHSV serotypes 1, 3 and 4 from a live-attenuated vaccine used in SA.

It is not possible to predict to what extent the characteristics of new emerging viruses can change and what the limitations of such changes will be. The new virus can be more pathogenic especially if reassortment results in reversion to virulence, can cross the placenta increasing its overwintering capabilities, or susceptibility to the vector resulting in more efficient spread of the virus (reviewed by Wilson, Darpel & Mellor 2008).

## Conclusion

The live-attenuated vaccine serotypes introduced by annual vaccination of BTV and the presence of the 21 endemic BTV serotypes in SA increase the co-circulation of viruses and provide multiple opportunities for the establishment of BTV reassortants (Gerdes 2004). The prolonged viraemia of BTV in cattle together with the fact that MLV BTV can replicate to titres high enough to infect *Culicoides* midges creates the perfect opportunity for emergence of new viruses by means of reassortment (Veronesi et al. 2005). Reassortment therefore contributes to the high heterogeneity of circulating BTVs and might be responsible for the large variation in virulence between field strains. Cattle, in particular, may facilitate this process because of their prolonged viraemia and sub-clinical disease presentation. Understanding the mechanisms and consequences of BTV reassortment will assist in the control and prevention of BT.

## References

[CIT0001] Attoui H., Billoir F., Cantaloube J.F., Biagini P., deMicc P. & De Lamballerie X, 2000, ‘Strategies for the sequence determination of viral dsRNA genomes’, *Journal of Virological Methods* 89, 147–158. https://doi.org/10.1016/S0166-0934(00)00212-3 10996648 10.1016/s0166-0934(00)00212-3

[CIT0002] Barnard B.J.H., Gerdes G.H. & Meiswinkel R, 1998, ‘Some epidemiological and economic aspects of a bluetongue-like disease in cattle in South Africa – 1995/96 and 1997’, *Onderstepoort Journal of Veterinary Research* 65(3), 145–151. 9809318

[CIT0003] Basak A.K., Grimes J.M., Gouet P., Roy P. & Stuart D.I, 1997, ‘Structures of orbivirus VP 7: Implications for the role of this protein in the viral life cycle’, *Structure* 5(7), 871–883. https://doi.org/10.1016/S0969-2126(97)00242-6 9261081 10.1016/s0969-2126(97)00242-6

[CIT0004] Batten C.A., Maan S., Shaw A.E., Maan N.S. & Mertens P.P, 2008, ‘A European field strain of bluetongue virus derived from two parental vaccine strains by genome segments reassortment’, *Virus Research* 137(1), 56–63. https://doi.org/10.1016/j.virusres.2008.05.016 18598726 10.1016/j.virusres.2008.05.016

[CIT0005] Boyce M., Celma C.C. & Roy P, 2012, ‘Bluetongue virus non-structural protein 1 is a positive regulator of viral protein synthesis’, *Virology Journal* 9, 178. 22931514 10.1186/1743-422X-9-178 PMC3479040

[CIT0006] Boyce M., Wehrfritz J., Noad R. & Roy P, 2004, ‘Purified recombinant bluetongue virus VP 1 exhibits RNA replicase activity’, *Journal of Virology* 78(8), 3994–4002. https://doi.org/10.1186/1743-422X-9-178 15047815 10.1128/JVI.78.8.3994-4002.2004 PMC374272

[CIT0007] Chauveau E., Doceul V., Lara E., Breard E., Sailleau C., Vidalain P.O. et al., 2013, ‘NS3 of bluetongue virus interferes with the induction of type I interferon’, *Journal of Virology* 87(14), 8241–8246. https://doi.org/10.1128/JVI.00678-13 23658442 10.1128/JVI.00678-13 PMC3700197

[CIT0008] Dungu B., Gerdes T. & Smit T, 2004, ‘The use of vaccination in the control of bluetongue in Southern Africa’, *Veterinaria Italiana* 40(4), 616–622. 20422597

[CIT0009] Elia G., Savini H., Decaro N., Martella V. Teodori l., Casaccia C. et al., 2008, ‘Use of real-time RT-PCR as a rapid molecular approach for differentiation of field and vaccine strains of bluetongue virus serotype 2 and 9’, *Molecular and Cellular Probes* 22(1), 38–46. https://doi.org/10.1016/j.mcp.2007.06.005 17693055 10.1016/j.mcp.2007.06.005

[CIT0010] Gerdes G.H, 2004, ‘A South African overview of the virus, vectors, surveillance and unique features of bluetongue’, *Veterinaria Italiana* 40(3), 39–42. 20419632

[CIT0011] Hassan S.S. & Roy P, 1999, ‘Expression and functional characterization of bluetongue virus VP 2 protein: Role in cell entry’, *Journal of Virology* 73(12), 9832–9842. 10559295 10.1128/jvi.73.12.9832-9842.1999 PMC113032

[CIT0012] Hassan S.H., Wirblich C., Forzan M. & Roy P, 2001, ‘Expression and functional characterization of bluetongue virus VP5 protein: Role in cellular permeabilization’, *Journal of Virology* 75(18), 8356–8367. https://doi.org/10.1128/JVI.75.18.8356-8367.2001 11507181 10.1128/JVI.75.18.8356-8367.2001 PMC115081

[CIT0013] Howell P.G., Alexander R.A., Clark R., Louw J.G. & De Kock V.E, 1960, ‘A preliminary antigenic classification of strains of bluetongue virus’, *Onderstepoort Journal of Veterinary Research* 28, 357–364, viewed 12 March 2015, from https://repository.up.ac.za/handle/2263/57072

[CIT0014] Howell P.G. & Verwoerd D.W, 1971, ‘Bluetongue virus’, *Virology Monographs* 9, 35–74. https://doi.org/10.1007/978-3-7091-3987-5_2 10.1007/978-3-7091-3987-5_2 4354734

[CIT0015] Hyatt A.D., Zhao Y. & Roy P, 1993, ‘Release of bluetongue virus-like particles from insect cells is mediated by BTV non-structural protein NS3/NS3A’, *Virology* 193(2), 592–603. https://doi.org/10.1006/viro.1993.1167 8384747 10.1006/viro.1993.1167

[CIT0016] Kar A.K., Bhattacharya B. & Roy P, 2007, ‘Bluetongue virus RNA binding protein NS2 is a modulator of viral replication and assembly’, *BMC Molecular Biology* 8(1), 4, viewed 25 March 2015, from https://www.ncbi.nlm.nih.gov/pubmed/17241458 17241458 10.1186/1471-2199-8-4 PMC1794256

[CIT0017] Maan N.S., Maan S., Nomikou K., Guimera M., Pullinger G., Singh K.P. et al., 2012, ‘The genome sequence of bluetongue virus type 2 from India: Evidence for reassortment between eastern and western topotype field strains’, *Journal of Virology* 86(10), 5967–5968. https://doi.org/10.1128/JVI.00536-12 22532533 10.1128/JVI.00536-12 PMC3347281

[CIT0018] Maan S., Maan N.S., Van Rijn P.A., Van Gennip R.G., Sanders A., Wright I.M. et al., 2010, ‘Full genome characterisation of bluetongue virus serotype 6 from the Netherlands 2008 and comparison to other field and vaccine strains’, *PLoS One* 5, e10323 https://doi.org/10.1371/journal.pone.0010323 10.1371/journal.pone.0010323 PMC2859060 20428242

[CIT0019] MacLachlan N.J., Nunamaker R.A., Katz J.B., Sawyer M.M., Akita G.Y., Osburn B.I. et al., 1994, ‘Detection of bluetongue virus in the blood of inoculated calves: Comparison of virus isolation, PCR assay, and *in vitro* feeding of *Culicoides variipennis*’, *Archives of Virology* 136(1–2), 1–8. https://doi.org/10.1007/BF01538812 8002778 10.1007/BF01538812

[CIT0020] Mellor P.S., Boorman J. & Baylis M, 2000, ‘*Culicoides* biting midges: Their role as arbovirus vectors’, *Annual Review of Entomology* 45(1), 307–340. https://doi.org/10.1146/annurev.ento.45.1.307 10.1146/annurev.ento.45.1.307 10761580

[CIT0021] Mertens P.P., Diprose J., Maan S., Singh K.P., Attoui H. & Samuel A.R, 2004, ‘Bluetongue virus replication, molecular and structural biology’, *Veterinaria Italiana* 40(4), 426–437. 20422565

[CIT0022] Modumo J. & Venter E.H, 2012, ‘Determination of the minimum protective dose for bluetongue virus serotype 2 and 8 vaccines in sheep’, *Journal of the South African Veterinary Association* 83(1), 1–6. https://doi.org/10.4102/jsava.v83i1.17 23327131 10.4102/jsava.v83i1.17

[CIT0023] Nomikou K., Hughes J., Wash R., Kellam P., Breard E., Zientara S. et al., 2015, ‘Widespread reassortment shapes the evolution and epidemiology of bluetongue virus following European invasion’, *PLoS Pathogens* 11(8), e1005056 https://doi.org/10.1371/journal.ppat.1005056 26252219 10.1371/journal.ppat.1005056 PMC4529188

[CIT0024] Potgieter A.C., Page N.A., Liebenberg J., Wright I.M., Landt O. & Van Dijk A.A, 2009, ‘Improved strategies for sequence-independent amplification and sequencing of viral double stranded RNA genomes’, *Journal of General Virology* 90, 1–11. https://doi.org/10.1099/vir.0.009381-0 19264638 10.1099/vir.0.009381-0

[CIT0025] Purse B.V., Mellor P.S. Rogers D.J. Samuel A.R. Mertens P.P. & Baylis M, 2005, ‘Climate change and the recent emergence of bluetongue in Europe’, *Nature Reviews Microbiology* 3, 171–181. https://doi.org/10.1038/nrmicro1090 15685226 10.1038/nrmicro1090

[CIT0026] Ramadevi N., Burroughs N.J., Mertens P.P., Jones I.M. & Roy P, 1998, ‘Capping and methylation of mRNA by purified recombinant VP4 protein of bluetongue virus’, *Proceedings of the National Academy of Sciences USA* 95, 13537–13542. https://doi.org/10.1073/pnas.95.23.13537 10.1073/pnas.95.23.13537 PMC24854 9811835

[CIT0027] Ramig R.F., Garrison C., Chen D. & Bell-Robinson D, 1989, ‘Analysis of reassortment and superinfection during mixed infection of Vero cells with bluetongue virus serotypes 10 and 17’, *Journal General Virology* 70, 2595–2603. https://doi.org/10.1099/0022-1317-70-10-2595 10.1099/0022-1317-70-10-2595 2552005

[CIT0028] Ratinier M., Caporale M., Golder M., Franzoni G., Allan K., Nunes S.F. et al., 2011, ‘Identification and characterization of a novel non-structural protein of bluetongue virus’, *PLoS Pathogenesis* 7(12), e1002477 https://doi.org/10.1371/journal.ppat.1002477 10.1371/journal.ppat.1002477 PMC3248566 22241985

[CIT0029] Samal S.K., el-Hussein A., Holbrook F.R., Beaty B.J. & Ramig R.F, 1987a, ‘Mixed infection of *Culicoides variipennis* with bluetongue virus serotypes 10 and 17: Evidence for high frequency reassortment in the vector’, *Journal General Virology* 68, 2319–2329. https://doi.org/10.1099/0022-1317-68-9-2319 10.1099/0022-1317-68-9-2319 2821173

[CIT0030] Samal S.K., Livingston C.W. Jr., McConnell S. & Ramig R.F, 1987b, ‘Analysis of mixed infection of sheep with bluetongue virus serotypes 10 and 17: Evidence for genetic reassortment in the vertebrate host’, *Journal of Virology* 61, 1086–1091. 3029402 10.1128/jvi.61.4.1086-1091.1987 PMC254067

[CIT0031] Savini G., MacLachlan N.J., Sánchez-Vizcaíno J.M. & Zientara S, 2008, ‘Vaccine against bluetongue in Europe’, *Comparative Immunology, Microbiology & Infectious Diseases* 31(2), 101–120. https://doi.org/10.1016/j.cimid.2007.07.006 10.1016/j.cimid.2007.07.006 17765305

[CIT0032] Sellers R.F. & Taylor W.P, 1980, ‘Epidemiology of bluetongue and the import and export of livestock, semen and embryos’, *Bulletin de I’Office International des Épizooties* 92, 587–592.

[CIT0033] Shafiq M., Minakshi P., Bhateja A., Ranjan K. & Prasad G, 2013, ‘Evidence of genetic reassortment between Indian isolate of bluetongue virus serotype 21 (BTV-21) and bluetongue serotype 16 (BTV-16)’, *Virus Research* 173, 336–343. https://doi.org/10.1016/j.virusres.2013.01.009 23353779 10.1016/j.virusres.2013.01.009

[CIT0034] Singer R.S., MacLachlan N.J. & Carpenter T.E, 2001, ‘Maximal predicted duration of viraemia in bluetongue virus–infected cattle’, *Journal of Veterinary Diagnostic Investigation* 13(1), 43–49. https://doi.org/10.1177/104063870101300109 11243362 10.1177/104063870101300109

[CIT0035] Stauber N., Martinez-Costas J., Sutton G., Monastyrskaya K. & Roy P, 1997, ‘Bluetongue virus VP6 protein binds ATP and exhibits an RNA-dependent ATPase function and a helicase activity that catalyze the unwinding of double-stranded RNA substrates’, *Journal of Virology* 71(10), 7220–7226. 9311795 10.1128/jvi.71.10.7220-7226.1997 PMC192062

[CIT0036] Steyn J., Coetzee P., Venter G. & Venter E.H, 2015, ‘The epidemiology of bluetongue virus in Mnisi, South Africa’, *American Journal of Epidemiology and Infectious Diseases* 3(5), 95–102.

[CIT0037] Stott J.L., Oberst R.D., Channell M.B. & Osburn B.I, 1987, ‘Genome segment reassortment between two serotypes of bluetongue virus in a natural host’, *Journal of Virology* 61(9), 2670–2674. 3039160 10.1128/jvi.61.9.2670-2674.1987 PMC255769

[CIT0038] Van den Bergh C., Coetzee P., Guthrie A.J., Le Grange M. & Venter E.H, 2016, ‘Complete genome sequences of five bluetongue virus (BTV) vaccine strains from a commercial live attenuated vaccine, a BTV-4 field strain from South Africa, and a reassortant strain isolated from experimentally vaccinated cattle’, *Genome Announcements* 4(3), e00462–16. https://doi.org/10.1128/genomeA.00462-16 27340051 10.1128/genomeA.00462-16 PMC4919390

[CIT0039] Veronesi E., Hamblin C. & Mellor P.S, 2005, ‘Live attenuated bluetongue vaccine viruses in Dorset poll sheep, before and after passage in vector midges (Diptera: Ceratopogonidae)’, *Vaccine* 23(48), 5509–5516. https://doi.org/10.1016/j.vaccine.2005.07.039 16111787 10.1016/j.vaccine.2005.07.039

[CIT0040] Weyer C.T., Grewar J.D., Burger P., Russouw E., Lourens C.W., Joone C. et al., 2017, ‘African horse sickness caused by genome reassortment and reversion to virulence of live, attenuated vaccine viruses, South Africa, 2004–2014’, *Emerging Infectious Diseases* 22(12), 2087–2096. https://doi.org/10.3201/eid2212.160718 10.3201/eid2212.160718 PMC5189153 27442883

[CIT0041] Wilson A., Darpel K. & Mellor P.S, 2008, ‘Where does bluetongue virus sleep in the winter?’, *PLoS Biology* 6(8), e210 https://doi.org/10.1371/journal.pbio.0060210 18752350 10.1371/journal.pbio.0060210 PMC2525685

